# Graft-versus-lymphoma effect in a 64-year-old caucasian woman after allogeneic stem-cell transplantation: a case report

**DOI:** 10.1186/1752-1947-3-10

**Published:** 2009-01-13

**Authors:** Gerhard Behre, Thomas Weber, Sebastian Theurich, Maximilian Christopeit

**Affiliations:** 1 Department of Internal Medicine IV, Oncology and Hematology, Martin-Luther-University Halle-Wittenberg, Ernst-Grube-Str. 40, D-06097 Halle, Germany

## Abstract

**Introduction:**

The existence of a graft-versus-lymphoma effect is well established. When lacking a firm diagnosis, however, the clinician is challenged to to weigh the potential benefits of the graft-versus-lymphoma effect against potential dangers of graft-versus-host disease as well as against generalized (viral) infections.

**Case presentation:**

We present evidence for a graft-versus-lymphoma effect in a 64-year-old caucasian woman with a transplanted peripheral blood-stem-cell graft from her Human Leukocyte Antigen-identical sister, and propose diagnostic measures to distinguish between graft-versus-host effect, and against viral disease or drug-induced reactions.

**Conclusion:**

We were able to identify an allogeneic graft-reaction against progressive lymphoma alongside an erythema consistent with acute graft-versus-host disease of the skin. Establishing a firm diagnosis enabled us to decide against T-cell suppression (such as by using cyclosporine). Anti-lymphoma activity was favoured, by means of the allogeneic graft, local radiation and immunotherapy. This illustrates the importance of a sound differential diagnosis of erythema after allogeneic stem-cell transplantation, including assessment of viral disease of the affected tissue.

## Introduction

The existence of a graft-versus-lymphoma effect is well established. Its clinical significance is less clear and seems to depend on the type of lymphoma and on the disease status [1]. A graft-versus-lymphoma effect frequently coincides with graft-versus-host disease. Assessment of graft-versus-host disease is commonly obscured by concomitant diseases, e.g. viral or drug-induced exanthema [2,3], viral hepatitis or viral gastroenteritis. The crucial decision is if, when and to which extent an immunosuppressant should be administered. Lacking a firm diagnosis, the challenge for the clinician is to outweigh potential benefits of the graft-versus-lymphoma effect against potential dangers of graft-versus-host disease as well as against generalized viral infections.

## Case presentation

**Figure 1:**
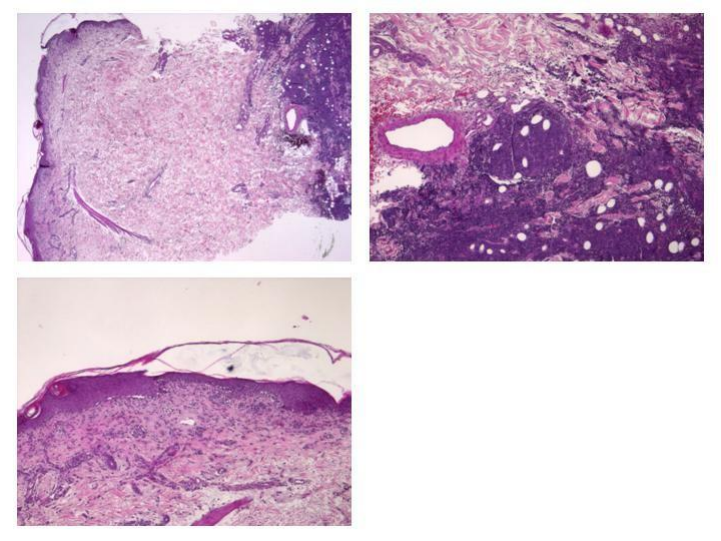
**Lymphoma infiltration spreading subcutaneously accompanied by a lichenoid inflammation**.

A 64-year-old female patient received a peripheral blood-stem-cell graft from her Human Leukocyte Antigen (HLA)-identical sister in the course of a refractory lymphoplasmacytic lymphoma. Conditioning consisted of 7 Gy total body irradiation (2 × 2 Gy d-8, 1 × 3 Gy d-7), 120 mg/m^2 ^fludarabin (30 mg/m^2 ^d-7 – d-4) and cyclophosphamide 60 mg/kg (30 mg/kg, d-4 – d-3). During conditioning, the lymphoma progressed and histology showed transformation into an aggressive lymphoma. In the course of the disease, the patient rapidly developed new small, callous subcutaneous nodules. Radiation as well as immunotherapy (anti-CD20-antibody, rituximab) was administered and immunosuppression discontinued. Unfortunately, growth of the lymphomatous lesions could not be halted by these means. After the patient had engrafted, a progressive erythema, predominantly surrounding the novel subcutaneous nodules, evolved and was followed by massive diarrhea, prompting the differential diagnosis of acute graft-versus-host disease versus viral disease. A skin biopsy in proximity to a subcutaneous nodule surprisingly not only showed a polymerase chain reaction (PCR) positive for Epstein-Barr Virus (EBV) but also histologic findings of both lymphoma and graft versus lymphoma (Figure 1). Unfortunately, the patient died of multiple organ failure following sepsis with multiple causative organisms, including EBV, Cytomegalovirus (CMV) and gram negative bacteria.

## Conclusion

To summarize, we were able to identify an allogeneic graft reaction against progressive lymphoma as the cause of the observed erythema. We therefore were able to decide against T-cell-suppression, for example by cyclosporine, favouring anti-lymphoma activity by means of the allogeneic graft, local radiation and immunotherapy. We were not able to establish the relevance of a positive EBV PCR detected in proximity to the subcutaneous nodules in the skin. Notably, the skin biopsies stained negative for latent membrane protein 1 (LMP1), making a post-transplantation lymphoproliferative disease less likely than a mere transformation of the indolent into an aggressive lymphoma.

This case report illustrates the importance of a sound differential diagnosis of erythema after allogeneic stem-cell transplantation, including assessment of viral disease of the affected tissue with PCR as well as histology.

## Abbreviations

HLA: Human Leukocyte Antigens; PCR: polymerase chain reaction; EBV: Epstein-Barr Virus; CMV: Cytomegalovirus; LMP1: latent membrane protein 1

## Consent

Written informed consent was obtained from the patient for publication of this case report and accompanying images. A copy of the written consent is available for review by the Editor-in-Chief of this journal.

## Competing interests

The authors declare that they have no competing interests.

## Authors' contributions

The authors all treated the patient and wrote the manuscript.
